# Unilateral percutaneous endoscopic debridement and drainage for lumbar infectious spondylitis

**DOI:** 10.1186/s13018-018-1009-5

**Published:** 2018-12-03

**Authors:** Xuepeng Wang, Shaobo Zhou, Zhenyu Bian, Maoqiang Li, Wu Jiang, Changju Hou, Liulong Zhu

**Affiliations:** Department of Orthopedics Surgery, Hangzhou First People’s Hospital, Zhejiang University School of Medicine, 261 Huansha Road, Hangzhou, 310006 Zhejiang People’s Republic of China

**Keywords:** Lumbar infectious spondylitis, Percutaneous endoscopic debridement, Negative-pressure drainage, Sensitive antibiotics, Physiological saline

## Abstract

**Background:**

The treatment of lumbar infectious spondylitis is controversial. In this study, we attempted to demonstrate that unilateral percutaneous endoscopic debridement with physiologic saline and negative pressure drainage postoperatively may achieve a satisfactory result in lumbar infectious spondylitis.

**Methods:**

We retrospectively analyzed 17 patients with lumbar infectious spondylitis who underwent percutaneous endoscopic debridement and drainage (PEDD) through a posterolateral transforaminal approach. Each biopsy specimen was submitted without delay after surgery and examined for microorganisms and evaluated histopathologically. Patients were assessed by careful physical examination, MacNab criteria, Oswestry Disability Index (ODI), visual analog scale (VAS), regular serological tests, imaging studies for clinical function, and patient satisfaction.

**Results:**

Of the 17 patients, 14 (82.4%) had satisfactory relief of their back pain according to MacNab criteria at 1 week after PEDD. Three patients (17.6%) who had advanced infections with multilevel involvement and paraspinal abscesses underwent anterior debridement and autograft interbody fusion with instrumentation within 2 weeks. However, there were no other severe surgery-related complications. Causative bacteria were identified in most cases, and *Staphylococcus aureus* was the most prevalent strain.

**Conclusions:**

Unilateral PEDD with physiological saline or empirical antibiotics did not disrupt lumbar stability and avoided the important intraspinal structures such as the dural sac and nerve roots. It not only had a high rate of identification of the causative pathogen, but also provided effective infection control and pain relief. PEDD may be a useful technique for treatment of lumbar infectious spondylodiscitis patients who have no severe deformities and are unable to undergo the conventional anterior surgery due to poor health or advanced age.

## Background

Infectious spondylitis and infectious discitis are rare diseases that can cause significant clinical problems, including spinal deformity and segmental instability. Infectious spondylitis is usually found in the lumbar spine, which can be divided into pyogenic (the most frequent), non-specific, and specific (such as tuberculosis) types [1]. It can spontaneously occur in immunocompromised patients as a result of hematogenous spread from other inflammatory foci or following diagnostic and operative procedures [2]. The typical clinical signs and symptoms of lumbar infectious spondylitis, either pyogenic or tuberculous, include severe back pain with or without paralysis. In fact, magnetic resonance imaging (MRI) can provide sufficient details for non-invasive diagnosis of lumbar infectious spondylitis, with an approximate sensitivity of 96% and specificity of 93% [3, 4]. Although laboratory and radiologic findings can also assist in making an early diagnosis, inappropriate therapeutic choices and delayed efficacious treatments have led to many failed clinical cases [5].

There are a number of treatment strategies for lumbar infectious spondylitis, such as conservative therapy, traditional open surgery, and minimally invasive surgery. Traditionally, conservative therapy with appropriate antibiotics and bedrest was thought to be adequate for most patients with infectious spondylitis [6]. Conservative treatment requires large doses of antibiotics until 2 weeks after all clinical symptoms have disappeared and erythrocyte sedimentation rate (ESR) and C-reactive protein (CRP) have returned to normal [7]. The goal of conservative treatment is to wait for natural bone fusion progression into the intervertebral region and achieve pain relief by avoiding activities that can contribute to the gap between the upper and lower lumbar vertebral bodies. This process often takes more than 3 months, which is difficult for patients and their families [8, 9]. Open spinal surgery consisting of anterior or posterior debridement and bone grafting with or without supplemental instrumentation often leads to undesired postoperative complications [10]. Inappropriate open surgery can damage the vulnerable spinal cord and nerve roots, disrupt spinal stability, and even inflict additional trauma on suffering patients.

Percutaneous endoscopic discectomy was first used in the early 1980s to treat uncomplicated herniated discs. It has recently been used in spinal stenosis cases with favorable results [11]. Some studies also reported that percutaneous endoscopic discectomy can be performed in the management of lumbar infections without severe neurological symptoms [12–14]. It has advantages over other minimally invasive surgeries for the treatment of lumbar infectious spondylitis, being able to help both diagnose and treat at the same time. Yang et al. [14, 15] performed bilateral portal percutaneous endoscopic debridement and lavage with a dilute povidone-iodine solution to effectively treat pyogenic spondylitis with a paraspinal abscess and recurrent postoperative infection. In this study, we attempted to demonstrate that unilateral percutaneous endoscopic debridement with physiological saline would be adequate treatment for lumbar infectious spondylitis with or without a paraspinal abscess, and that appropriate antibiotic administration and negative pressure drainage postoperatively could achieve a satisfactory outcome.

## Methods

### Study population

Seventeen patients diagnosed with lumbar infectious spondylitis between January 2014 and July 2017 were enrolled in this study. The participants included 6 women and 11 men, with an average age of 59.5 years (range, 37–83 years). The affected levels ranged from L1/2 to L5/S1. Single level infection was seen in 12 patients, 1 with L1/2, 3 with L2/3, 2 with L3/4, 3 with L4/5, and 3 with L5/S1. Multilevel infection was seen in five cases, three with L4/5 and L5/S1 levels, and two with L2/3 and L3/4 levels. As for the Griffiths classification, there were four patients in class 1, 11 in class 2, and 2 in class 3. As for the Kulowski classification, there were five patients in acute, eight in subacute, and 4 in Latent. To reflect the friable conditions of enrolled patients, only patients with spontaneous spondylitis were enrolled, or those with iatrogenic spondylitis. Our indications for performing percutaneous endoscopic debridement and drainage (PEDD) included (1) intolerable back and/or radiating pain caused by lumbar infectious spondylitis; (2) elevated ESR and CRP values; and (3) radiographic film and MRI findings, namely, narrowing of the intervertebral disc space and variable degrees of destruction of the adjacent vertebral endplates, particularly disc hyperintensity on T2-weighted MRI imaging. Patients with progressive neurologic deficit due to epidural abscesses or spinal instability caused by significant structural destruction were excluded. Patients who had a history of spinal surgery were also excluded from the study.

**Fig. 1:**
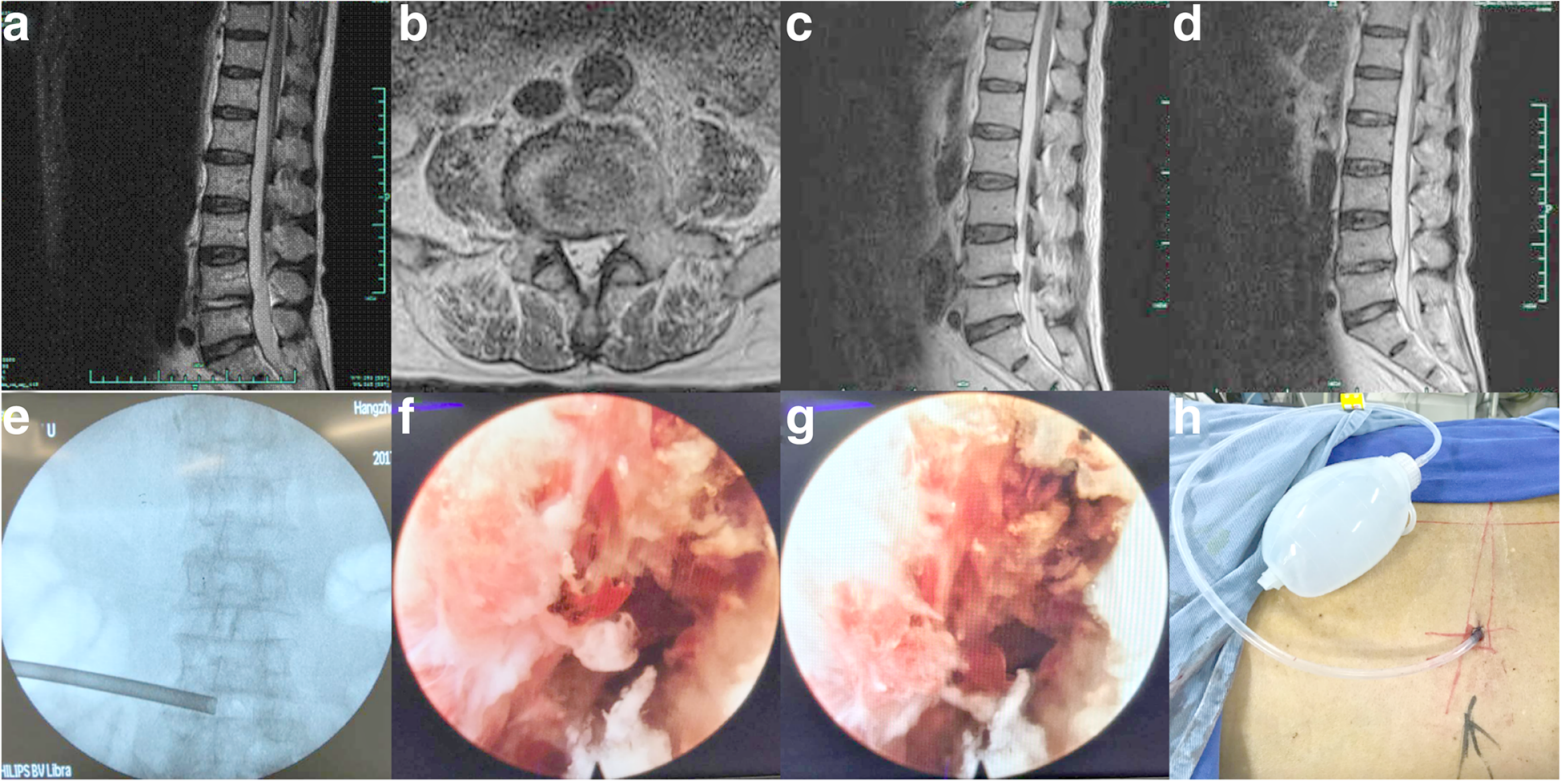
Typical case (case number 2). A 71-year-old man was diagnosed with having L5–S1 infectious spondylitis. A sagittal and axial T2-weighted MRI revealed L5–S1 infection with a paraspinal abscess (**a**, **b**). Unilateral percutaneous endoscopic debridement and drainage was performed (**e**, **h**). On endoscopic views, after the necrotic tissue and pus were discharged, the chapped disc was evident (**f**, **g**). Postoperative sagittal T2-weighted MRI at the 1 week (**c**) and 1 month (**d**) follow-ups demonstrated a decrease in the abnormally high signal in the L5/S1 intervertebral disc and a disappearance of the abscess.

The electronic medical records of the patients were reviewed thoroughly. The microbiology reports included microscopy and culture findings, and any specific pathogens identified by the PEDD procedures. All patients in this study presented with intractable back pain requiring narcotic pain control and bed rest before PEDD. In fact, empirical antibiotics were started with initial diagnoses of infective spondylitis. In most of patients, a failure of 2–4 weeks conservative treatment course (rest, waistband, antibiotics) had been taken before PEDD. Two patients who were suspected to have Mycobacterium tuberculosis were continued on antituberculous therapy for at least 2 weeks prior to PEDD. The antibiotic therapy regimen should be changed after PEDD according to the tissue cultures obtained intraoperatively and the corresponding antimicrobial susceptibility test. Appropriate antibiotics should be continued for at least 6 weeks in all patients postoperatively (Fig. 1).

### Surgical technique

The transforaminal endoscopic procedure was performed at levels where infection was observed on preoperative MRI scans. A posterolateral approach similar to the Yeung endoscopic technique with an inside-out technique was adopted for our spinal needle placement, although we used the Maxmorespine® surgical instrument system invented by Thomas Hoogland. The patients were positioned prone on a radiolucent frame suitable for intraoperative fluoroscopy. Sterile preparation and draping were performed, local anesthesia was achieved with 1% lidocaine, and a spinal needle was inserted directly into the targeted disc through Kambin’s triangle. Under fluoroscopic guidance, the spinal needle was inserted to the outer fibers of the annulus in a trajectory 15–30° from the sagittal plane and at a point 8–12 cm from the midline. The optimal insertion point was the line at the center of the pedicles on anteroposterior imaging, and the line at the posterior edge of the lumbar bodies on lateral imaging. A guide wire was introduced into the disc space through the spinal needle, and the spinal needle was subsequently withdrawn. The skin was cut off 0.5 cm from the center of the guide wire, and a tissue dilator was inserted over the wire down to the annulus. The tissue dilator was removed, and the manual cannulated drills were inserted into the target foramina. In some cases, a foraminoplasty was necessary before the working sleeve was inserted. The endoscope was then passed through the cannulated sleeve, and under saline irrigation, the infected disc structures were visible on the camera monitor. Micro forceps were then inserted through the sleeve to extract specimen from the infected disk for later microbiological and pathological testing. In order to improve the sensitivity of bacterial biopsy, the specimen could be also harvested by forceps before inserting the sleeve. Percutaneous debridement was performed piecemeal by manipulating the forceps, flexible rongeurs, and bipolar coagulation into different positions to withdraw as much necrotic tissue as possible. After the debridement procedures, about 1000 mL of physiological saline was used for irrigation. If needed, broad-spectrum antibiotics were injected into the affected area. Finally, one tube was inserted into the debrided disc space and connected to a negative-pressure drain after the wound was sutured closed. Appropriate antibiotics were administrated into the debrided disc space through the tube for at least three consecutive days after surgery before the negative-pressure drainage tube was removed.

### Outcome assessment

Their clinical outcomes were assessed by careful physical examination, MacNab criteria, Oswestry Disability Index (ODI), visual analog scale (VAS), regular serological tests, and imaging studies before surgery, and at 1 day, 1 week, 1 month, and 3 months postoperatively to determine whether open surgical intervention was necessary. Each biopsy specimen from patients was submitted without delay after PEDD and was examined for microorganisms and evaluated histopathologically. These outcomes before and after PEDD at different time points were compared using paired *t* tests. A value of *p* < 0.05 was considered statistically significant. SPSS 13.0 software (SPSS Inc., Chicago, IL, USA) was used for data analysis.

## Results

The most prominent clinical sign of infectious spondylitis was back pain, which was detected in all 17 patients before PEDD. In most cases, low back pain was relieved immediately after the operation. The mean operative time was 63.5 ± 16.7 min (range 40–120 min), the range of intraoperative blood loss was 10–20 ml, mean early ambulation occurred at 27.6 ± 8.3 h (range 4–48 h), and the mean length of hospital stay was 4.3 ± 1.9 days (range 3–10 days). As shown in Table 1, the VAS score, white blood cell (WBC), CRP, ESR, and neutrophilic granulocyte values at 1 day, 1 week, 1 month, and 3 months after surgery were lower than those before surgery (*p* < 0.05). The ODI at 3 months postoperatively was lower than it was before surgery (*p* < 0.05).

**Table 1:** Clinical outcomes and serological detections before and after percutaneous endoscopic debridement and drainage in successfully treated patients (*N* = 14, mean ± SD)

Parameter	Pre-OP	1 day post-OP	1 week post-OP	1 month post-OP	3 months post-OP
White blood cell (WBC) (× 10^9^/L)	14.56 ± 2.23	12.56 ± 1.87	8.68 ± 1.62	5.73 ± 1.34	5.36 ± 1.58
Neutrophilic granulocyte (%)	83.55 ± 10.24	78.87 ± 8.75	72.56 ± 6.73	52.67 ± 9.65	48.23 ± 5.28
Erythrocyte sedimentation rate (ESR)(mm/h)	63.55 ± 16.24	57.12 ± 14.26	41.84 ± 17.42	23.52 ± 4.23	11.22 ± 2.72
C-reactive protein (CRP) (mg/L)	83.67 ± 5.42	75.69 ± 8.18	42.73 ± 8.54	8.12 ± 3.87	2.63 ± 1.23
Visual analog scale (VAS)	7.67 ± 1.28	3.51 ± 0.83	2.35 ± 0.47	2.05 ± 0.86	1.05 ± 0.61
Oswestry Disability Index (ODI)	75.28 ± 13.53	–	–	–	16.23 ± 8.62

At 1 week after PEDD, 14 (82.4%) of the 17 patients reported satisfactory relief of their back pain according to MacNab criteria, including 5 who had excellent outcomes and 9 who had good outcomes (Table 2). The remaining three patients, including two with a fair outcome and one with a poor outcome, all had advanced infection with multilevel involvement, with paraspinal abscesses and severe back pain. Two of the 3 patients also had intermittent paresthesias of the lower limbs after PEDD. They underwent anterior debridement accompanied by autograft interbody fusion with instrumentation within 2 weeks after the PEDD, and this resulted in imperfect outcomes. However, no other severe surgery-related complications occurred in the other cases after PEDD.

**Table 2:** Patient demographic data and clinical outcomes

Case no.	Age (years)	Gender	Infection level	Use of immunosuppressant	Preoperative duration(m)	Kulowski classification	Griffiths classification	Diagnosis	MacNab criteria	Culture	Open surgery	Complication
1	51	F	L5/S1	No	3	Subacute	I	Postoperative infection	Good	ORSA	None	None
2	71	M	L4/5	Yes	2	Subacute	II	Infection with paraspinal abscess	Excellent	ORSA	None	None
3	66	M	L2/3,L3/4	No	0	Acute	II	Multilevel infection with paraspinal abscess	Fair	*Escherichia coli*	2 weeks later	None
4	53	M	L3/4	No	4	Subacute	II	Single level infection	Excellent	Streptococcus viridans	None	None
5	64	F	L4/5,L5/S1	No	13	Acute	II	Multilevel infection with epidural and paraspinal abscesss	Poor	Mycobacterium tuberculosis	2 weeks later	Paresthesia
6	83	M	L2/3	Yes	5	Acute	I	Single level infection	Good	OSSA	None	None
7	58	M	L4/5	No	0	Subacute	II	Single level infection	Excellent	Streptococcus pneumoniae	None	None
8	75	F	L4/5	Yes	1	Subacute	II	Postoperative infection	Good	*Staphylococcus epidermidis*	None	None
9	46	M	L2/3	No	7	Latent	II	Infection with epidural abscess	Good	ORSA	None	None
10	73	M	L4/5,L5/S1	No	21	Latent	III	Multilevel infection with paraspinal abscess	Fair	Pseudomonas aeruginosa	1 week later	Paresthesia
11	43	M	L2/3,L3/4	No	3	Subacute	II	Multilevel infection	Good	No growth	None	None
12	37	F	L1/2	No	6	Latent	II	Single level infection	Excellent	No growth	None	None
13	67	M	L5/S1	No	1	Subacute	I	Postoperative infection	Good	Streptococcus viridans	None	None
14	56	M	L4/5,L5/S1	Yes	7	Acute	II	Multilevel infection	Good	Enterococcus faecalis	None	None
15	49	M	L5/S1	No	5	Latent	III	Infection with paraspinal abscess	Good	Mycobacterium trberculosis	None	None
16	63	F	L2/3	No	0	Subacute	II	Single level infection	Excellent	No growth	None	None
17	57	F	L3/4	No	8	Acute	I	Single level infections	Good	ORSA	None	None

*M* male, *F* female, *L* lumbar spine, *S* sacral spine, *OSSA* oxacillin-sensitive *Staphylococcus aureus*, *ORSA* oxacillin-resistant *Staphylococcus aureus*

Causative bacteria were identified in the biopsy specimens of 14 (82.4%) of the 17 patients. As shown in Table 2, *Staphylococcus aureus* was the most prevalent stain, four of which were oxacillin-resistant, and one of which was oxacillin-sensitive. *Streptococcus viridans* and *Mycobacterium tuberculosis* were identified in one case each. The other five microbial strains identified included *Escherichia coli, Pseudomonas aeruginosa, Staphylococcus epidermidis, Streptococcus pneumoniae,* and *Enterococcus faecalis*. According to the culture and drug-susceptibility tests, intravenous infusions of appropriate antibiotics were given for at least 6 weeks after surgery. Two cases with *Mycobacterium tuberculosis* went on to receive antituberculous treatment after PEDD or two-stage operative anterior debridement.

## Discussion

The incidence of lumbar infectious spondylitis is low, but when it occurs, it is disastrous for patients and can lead to bone destruction, spinal deformities, and even paralysis. Early diagnosis and treatment of this disease is difficult, especially for low-toxicity bacterial infections. One of the reasons for this is the difficulty in detecting the organisms of spinal infection through blood cultures. There are reports that only half of blood cultures are positive in patients with lumbar infectious spondylitis [16]. The most reliable tests for finding the causative infectious agents are histological examinations and cultures of the samples taken from the infection sites. There were 17 cases of infectious spondylitis in the current study, including 2 patients with a specific infection (*Mycobacterium tuberculosis*) and 12 patients with a non-specific pyogenic infection. According to the patients’ medical histories, most cases were due to secondary infection, especially hematogenous dissemination. Besides these 14 identified cases, there were 3 cases with negative biopsy culture results, likely because of the preoperative use of antibiotics, insufficient biopsy using endoscopy, blended bacterial pollution, and the laboratory conditions to sensitive strains. Among the identified cases, except for 2 cases of *Mycobacterium tuberculosis*, *Staphylococcus aureus* was the most common strain (5/14), the isolation rate of which was consistent with previous reports such as that by Patel et al. [17]. In fact, *Staphylococcus aureus* has been reported to be present in up to 80% of cases of lumbar infectious spondylitis [17, 18]. Moreover, it was demonstrated that a great number of cases of infectious spondylitis had negative cultures, as shown in our study. Using different kinds of minimally invasive methods, the rates of bacterial detection have been reported to be 0.5–3.4% [19, 20]. Therefore, although bacterial culture is the gold standard for the diagnosis of infectious spondylitis, it is not necessary for early diagnosis and prompt treatment. Once lumbar infectious spondylitis is suspected, broad-spectrum antibiotics should be initiated immediately.

The most typical clinical manifestation of lumbar infectious spondylitis is intractable back pain, which worsens due to slight changes in position or vibration, and is often not alleviated by bed rest. Most patients have low-grade fever or no fever, and only a minority exhibits significant neurologic manifestations. ESR and CRP were shown to be significantly more reliable than WBC, and the total number of neutrophilic granulocytes in the identification of pyogenic spondylitis [21]. Elevated ESR within 6 weeks may be more meaningful than CRP for early diagnosis [22]. Regarding imaging, in most cases, spine radiographs remain within normal limits even after prolonged disease. Abnormalities may be seen on CT scans 3 weeks after the onset of illness, namely, narrowing of the intervertebral disc space and variable degrees of destruction of the adjacent vertebral endplates [23]. However, the most sensitive imaging method is MRI, which is currently considered the method of choice for diagnosis [24]. The characteristic images of infectious spondylitis on MRI are disc hypointensity on T1-weighted imaging, disc hyperintensity on T2-weighted imaging, or disc enhancement. Paravertebral or epidural abscesses show more diagnostic significance [25]. It is not difficult to confirm the diagnosis in patients with MRI signal changes in the intervertebral spaces, elevated ESR and CRP values, and low back pain. As shown in our study, three patients had negative biopsy culture results after surgery. Even if the postoperative bacterial culture is negative, this does not necessarily point to the presence of an aseptic degenerative disease such as discogenic back pain as opposed to lumbar infectious spondylitis.

The treatment of lumbar infectious spondylitis is controversial [26], and conservative versus operative treatment is frequently debated. Conservative treatment includes antimicrobial therapy, absolute bed rest, and other symptomatic treatments. Some scholars believe that the majority of infectious spondylitis cases can be cured by proper antimicrobial therapy, but no official guideline for antibiotics is available [27]. Other scholars have recently advocated surgical treatment, which may be able to clear the focal lesion and reconstruct the stability of the spine promptly [28]. Conservative treatment often takes more than 3 months while awaiting natural bony fusion of the intervertebral spaces, which is a long and difficult process for both patients and their families [29]. However, traditional open surgery brings and creates iatrogenic trauma, spinal instability, and other surgery-related complications such as spinal cord and nerve root injuries [30]. Therefore, an increasing number of patients have begun to accept minimally invasive and endoscopic surgery.

Under the endoscope working sleeve, a large amount of sterile physiological saline was used to lavage the lesion repeatedly after adequate debridement of the infected disc. This method can avoid dural tears and nerve roots injuries, and leads to minimal operative damage. Yang et al. [13] reported a group of cases using diluted povidone-iodine solution in minimally invasive endoscopic surgery for lumbar infectious spondylitis. In our study, we used sterile physiological saline instead, which also provided satisfactory relief of back pain and infection control without any other complications. Moreover, unlike their method, we used a unilateral working sleeve for endoscopic decompression, clearance, and drainage. Postoperative lavage using a drainage tube and broad-spectrum antibiotics or antituberculosis drugs was required, which was the preliminary step to ensure the effectiveness of PEDD. Compared with bilateral debridement and drainage via endoscopy as described by Yang et al. [13, 14], unilateral percutaneous endoscopic technique was adequate in our current study. The time needed to place a unilateral working sleeve was shorter than for bilateral sleeves, and it created less damage, while the function of unilateral drainage postoperatively may be similar to bilateral drainage. Patients treated with our present method had decreased VAS scores and improved ODI scores postoperatively. Neutrophil counts, and WBC, ESR, CRP values at different time points postoperatively were all significantly lower than before surgery. At 1 week after surgery, a good modified MacNab rating occurred in 82.2% of cases, which illustrated that the therapeutic effect of PEDD was satisfactory.

We believe that the reasonable assessment of patients preoperatively is the key to a successful operation. For a single segment of infectious spondylitis, PEDD can achieve satisfactory results by minimally invasive debridement combined with postoperative lavage and drainage. Even with the presence of paravertebral or epidural abscess, or in cases of tuberculosis, as long as there is no obvious spinal instability and no neurological symptoms, this less invasive method should be considered. These single-segment lumbar infections without severe nerve compression and kyphotic deformity are thought to be the best indication for PEDD. The three cases of failure in our study were all multi-segment infections, which were usually combined with a paravertebral abscess. Our minimally invasive treatment method was likely inadequate for the debridement of the multilevel infections. The postoperative lavage and vacuum suction were also inadequate to ensure the effective drainage of the paravertebral abscesses. The failures may not have been related to the invasive strength of the bacteria.

For the rare cases of specific infection or tuberculosis (2/17), the local postoperative drainage of streptomycin was important. In our study, the patient with a single-level tuberculous infection received satisfactory treatment using the above procedure. The other patient with a multilevel tuberculous infection and multiple paravertebral abscesses required an additional anterior open surgery. Therefore, for patients with tuberculous discitis or spondylitis, minimally invasive debridement and drainage may be an option prior to anterior decompression and instrumented fusion [31]. As mentioned above, lumbar infectious spondylitis occurs rarely. This study was limited by the small number of cases, the results of which may not be strongly recommended. It was also a retrospective non-controlled trial, with a lack of randomly assigned subjects or reasonable controls. If possible, more cases with infectious spondylitis, especially tuberculous spondylitis, should be studied to determine the true effect of PEDD. Moreover, the possibility that a second PEDD may be adequate instead of an open remedial surgery in cases with invasive bacterial infections also needs to be confirmed in future studies.

## Conclusions

Unilateral PEDD for lumbar infectious spondylitis does not disrupt lumbar stability, avoids important intraspinal structures such as the dural sac and the nerve roots, and reduces operative risks. Endoscopically, a large amount of physiological saline and empirical antibiotics are used to lavage the infected area until the lesions are removed completely through the intervertebral spaces. This kind of endoscopic surgery not only has a high rate of identifying the causative pathogen, but also provides effective infection control and pain relief, even if the causative pathogen cannot be identified. Sensitive antibiotics are delivered directly to the infected lesions for a few days following the PEDD procedure. Vacuum pressure is used to ensure effective drainage so that the infection can be quickly eradicated. We believe that unilateral PEDD is a useful technique for the treatment of lumbar infectious spondylodiscitis patients who have no severe deformity or nerve injury, and who are unable to undergo conventional anterior surgery due to having poor health or advanced age, or being immunocompromised.

## Abbreviations

CRP: C-reactive protein
ESR: Erythrocyte sedimentation rate
MRI: Magnetic resonance imaging
ODI: Oswestry Disability Index
PEDD: Percutaneous endoscopic debridement and drainage
VAS: Visual analog scale

## References

[1] Principi N, Esposito S (2016). Infectious discitis and spondylodiscitis in children. Int J Mol Sci.

[2] Chung TC, Yang SC, Chen HS (2014). Single-stage anterior debridement and fibular allograft implantation followed by posterior instrumentation for complicated infectious spondylitis: report of 20 cases and review of the literature. Medicine.

[3] Dunbar JA, Sandoe JA, Rao AS (2010). The MRI appearances of early vertebral osteomyelitis and discitis. Clin Radiol.

[4] Goethem JWMV, Parizel PM, Hauwe LVD (2000). The value of MRI in the diagnosis of postoperative spondylodiscitis. Neuroradiology.

[5] Kim JH, Kim MJ, Koh SE (2013). Thoracic infectious spondylitis after surgical treatments of herniated lumbar intervertebral disc. Ann Rehabil Med.

[6] Nickerson EK, Sinha R. Vertebral osteomyelitis in adults: an update. Br Med Bull. 2016;117(1):121–38. 10.1093/bmb/ldw003 26872859

[7] Valancius K, Hansen ES, Høy K (2013). Failure modes in conservative and surgical management of infectious spondylodiscitis. Eur Spine J.

[8] Fukuda K, Miyamoto H, Uno K (2014). Indications and limitations of conservative treatment for pyogenic spondylitis. J Spinal Disord Tech.

[9] Alton TB, Patel AR, Bransford RJ (2015). Is there a difference in neurologic outcome in medical versus early operative management of cervical epidural abscesses?. Spine J.

[10] Jr CD, Chittiboina P, Caldito G (2013). Comparison of operative and nonoperative management of spinal epidural abscess: a retrospective review of clinical and laboratory predictors of neurological outcome. J Neurosurg Spine.

[11] Wu B, Zhang S, Lian Q (2017). Lumbar scoliosis combined lumbar spinal stenosis and herniation diagnosed patient was treated with “U” route transforaminal percutaneous endoscopic lumbar discectomy. Case Rep Orthop.

[12] Ito M, Abumi K, Kotani Y (2007). Clinical outcome of posterolateral endoscopic surgery for pyogenic spondylodiscitis: results of 15 patients with serious comorbid conditions. Spine.

[13] Yang SC, Fu TS, Chen HS (2014). Minimally invasive endoscopic treatment for lumbar infectious spondylitis: a retrospective study in a tertiary referral center. BMC Musculoskelet Disord.

[14] Yang SC, Chen WJ, Chen HS (2014). Extended indications of percutaneous endoscopic lavage and drainage for the treatment of lumbar infectious spondylitis. Eur Spine J.

[15] Hsu LC, Tseng TM, Yang SC (2015). Bilateral portal percutaneous endoscopic debridement and lavage for lumbar pyogenic spondylitis. Orthopedics.

[16] Iwata A, Ito M, Abumi K (2014). Fungal spinal infection treated with percutaneous posterolateral endoscopic surgery. J Neurol Surg A Cent Eur Neurosurg.

[17] Patel AR, Alton TB, Bransford RJ (2014). Spinal epidural abscesses: risk factors, medical versus surgical management, a retrospective review of 128 cases. Spine J.

[18] Quiñoneshinojosa A, Jun P, Jacobs R (2004). General principles in the medical and surgical management of spinal infections: a multidisciplinary approach. Neurosurg Focus.

[19] Cornett CA, Vincent SA, Crow J (2016). Bacterial Spine Infections in Adults: Evaluation and Management. J Am Acad Orthop Surg.

[20] Kayser R, Mahlfeld K, Greulich M (2005). Spondylodiscitis in childhood: results of a long-term study. Spine.

[21] Chaudhary SB, Vives MJ, Basra SK (2007). Postoperative spinal wound infections and postprocedural diskitis. J Spinal Cord Med.

[22] Spencer SJ, Wilson NI (2012). Childhood discitis in a regional children’s hospital. J Pediatr Orthop B.

[23] Spencer SJ, Wilson N (2012). Discitis in childhood. Acta Orthop.

[24] Patel KB, Poplawski MM, Pawha PS (2014). Diffusion-weighted MRI “claw sign” improves differentiation of infectious from degenerative modic type 1 signal changes of the spine. AJNR Am J Neuroradiol.

[25] Daghighi MH, Poureisa M, Safarpour M (2016). Diffusion-weighted magnetic resonance imaging in differentiating acute infectious spondylitis from degenerative Modic type 1 change; the role of b-value, apparent diffusion coefficient, claw sign and amorphous increased signal. Br J Radiol.

[26] Fu TS, Yang SC, Tsai TT (2010). Percutaneous endoscopic debridement and drainage in immunocompromised patients with complicated infectious spondylitis. Minim Invasive Ther Allied Technol.

[27] Siam AE, Saghir HE, Boehm H (2016). Adjacent segment infection after surgical treatment of spondylodiscitis. J Orthop Traumatol.

[28] Zarghooni K, Röllinghoff M, Sobottke R (2012). Treatment of spondylodiscitis. Int Orthop.

[29] Wang YC, Wong CB, Wang IC (2016). Exposure of prebiopsy antibiotics influence bacteriological diagnosis and clinical outcomes in patients with infectious spondylitis. Medicine.

[30] Shousha M, Heyde C, Boehm H (2015). Cervical spondylodiscitis: change in clinical picture and operative management during the last two decades. A series of 50 patients and review of literature. Eur Spine J.

[31] Shibuya S, Komatsubara S, Yamamoto T (2009). Percutaneous discectomy-continuous irrigation and drainage for tuberculous lumbar spondylitis: a report of two cases. Case Rep Med.

